# IMSindel: An accurate intermediate-size indel detection tool incorporating *de novo* assembly and gapped global-local alignment with split read analysis

**DOI:** 10.1038/s41598-018-23978-z

**Published:** 2018-04-04

**Authors:** Daichi Shigemizu, Fuyuki Miya, Shintaro Akiyama, Shujiro Okuda, Keith A Boroevich, Akihiro Fujimoto, Hidewaki Nakagawa, Kouichi Ozaki, Shumpei Niida, Yonehiro Kanemura, Nobuhiko Okamoto, Shinji Saitoh, Mitsuhiro Kato, Mami Yamasaki, Tatsuo Matsunaga, Hideki Mutai, Kenjiro Kosaki, Tatsuhiko Tsunoda

**Affiliations:** 1 Department for Medical Genome Sciences, Medical Genome Center, National Center for Geriatrics and Gerontology, Aichi, Japan; 2 Department of Medical Science Mathematics, Medical Research Institute, Tokyo Medical and Dental University (TMDU), Tokyo, Japan; 3 RIKEN Center for Integrative Medical Sciences, Yokohama, Japan; 4 Medical Sciences Innovation Hub Program, Cluster for Science and Technology Hub, RIKEN, Yokohama, Japan; 5 CREST, JST, Japan; 6 Niigata University Graduate School of Medical and Dental Sciences, Niigata, Japan; 7 Department of Drug Discovery Medicine, Graduate School of Medicine, Kyoto University, Kyoto, Japan; 8 Division of Regenerative Medicine, Institute for Clinical Research, Osaka National Hospital, National Hospital Organization, Osaka, Japan; 9 Department of Neurosurgery, Osaka National Hospital, National Hospital Organization, Osaka, Japan; 10 Department of Medical Genetics, Osaka Medical Center and Research Institute for Maternal and Child Health, Osaka, Japan; 11 Department of Pediatrics and Neonatology, Nagoya City University Graduate School of Medical Sciences, Nagoya, Japan; 12 Department of Pediatrics, Showa University School of Medicine, Tokyo, Japan; 13 Department of Pediatric Neurosurgery, Takatsuki General Hospital, Osaka, Japan; 14 Division of Hearing and Balance Research, National Institute of Sensory Organs, National Hospital Organization Tokyo Medical Center, Tokyo, Japan; 15 Center for Medical Genetics, Keio University School of Medicine, Tokyo, Japan

## Abstract

Insertions and deletions (indels) have been implicated in dozens of human diseases through the radical alteration of gene function by short frameshift indels as well as long indels. However, the accurate detection of these indels from next-generation sequencing data is still challenging. This is particularly true for intermediate-size indels (≥50 bp), due to the short DNA sequencing reads. Here, we developed a new method that predicts intermediate-size indels using BWA soft-clipped fragments (unmatched fragments in partially mapped reads) and unmapped reads. We report the performance comparison of our method, GATK, PINDEL and ScanIndel, using whole exome sequencing data from the same samples. False positive and false negative counts were determined through Sanger sequencing of all predicted indels across these four methods. The harmonic mean of the recall and precision, F-measure, was used to measure the performance of each method. Our method achieved the highest F-measure of 0.84 in one sample, compared to 0.56 for GATK, 0.52 for PINDEL and 0.46 for ScanIndel. Similar results were obtained in additional samples, demonstrating that our method was superior to the other methods for detecting intermediate-size indels. We believe that this methodology will contribute to the discovery of intermediate-size indels associated with human disease.

## Introduction

A key aspect of genomic research is to determine the genetic difference among individuals and to understand the relationship between their phenotypic differences. Genomic variation is composed of single nucleotide polymorphisms (SNPs) and structural variations (SVs), such as insertions/deletions (indels) and duplications. Currently, a number of sophisticated computational approaches have been developed to accurately detect SNPs and short indels (<50 bp) from next-generation sequencing (NGS) data^1–3^. Large-scale SVs, including duplications, are not generally detected using NGS data, but they have been identified using the microarray technology arrayCGH at kilo-bases resolution^4–7^. Between these two size groups lies intermediate indels (50 bp to 10,000 bp), which are known to exist in the human genome^8^, but current detection methods using traditional NGS short read data still lack accuracy.

NGS short reads are generally aligned with a gapped aligner, such as BWA-MEM^9^, and the presence of indels are inferred. Such an approach is suitable for the detection of short indels, but is not applicable to the detection of intermediate-size indels because much of the information of these indels is lost in unmatched fragments of partially mapped reads. Several tools have been developed to detect these intermediate-size indels. They can mainly be classified into three approaches: (1) realignment based approach (GATK^1^, Scalpel^10^, SV-STAT^11^), (2) split-read approach (PINDEL^12^, Splitread^13^, PRISM^14^), and (3) local assembly approach (SOAPindel^15^). Recently, hybrid approaches that integrate these approaches have also been developed, resulting in more sensitive indel discovery methods than these approaches independently (ScanIndel^16^).

Here, we introduce a new method that detects InterMediate-Size indels using a combination of soft-clipped fragments realignment and *de novo* assembly of unmapped reads (IMSindel). We compare the performance of our method with three existing methods: GATK HaplotypeCaller^1^, which detects intermediate-size indels using a realignment based approach, PINDEL^12^, which uses a split-read approach, and ScanIndel^16^, which implements a hybrid approach, using whole exome sequencing (WES) data from three HapMap-JPT samples. Furthermore, we apply our method to actual disease samples with WES, and report on the size distribution of the intermediate-size indels predicted. This program, “genotype caller for InterMediate-Size indel (IMSindel)”, is publicly available at https://github.com/NCGG-MGC/IMSindel.

## Results

### Sequencing and mapping

We sequenced two individuals (NA18943 and NA18948) using the Illumina HiSeq. 2500 platform with paired-end reads of 161 bp. Mapping of the sequenced reads was performed using the short read mapping algorithm BWA-MEM^9^; 99.92% and 99.91% of WES reads were mapped to the human reference genome in NA18943 and NA18948, respectively. The PCR duplication rates, estimated using the Picard toolkit (http://broadinstitute.github.io/picard/), were 14.50% and 18.02% in NA18943 and NA18948, respectively.

### Summary for our intermediate-size indel prediction

**Figure 1:**
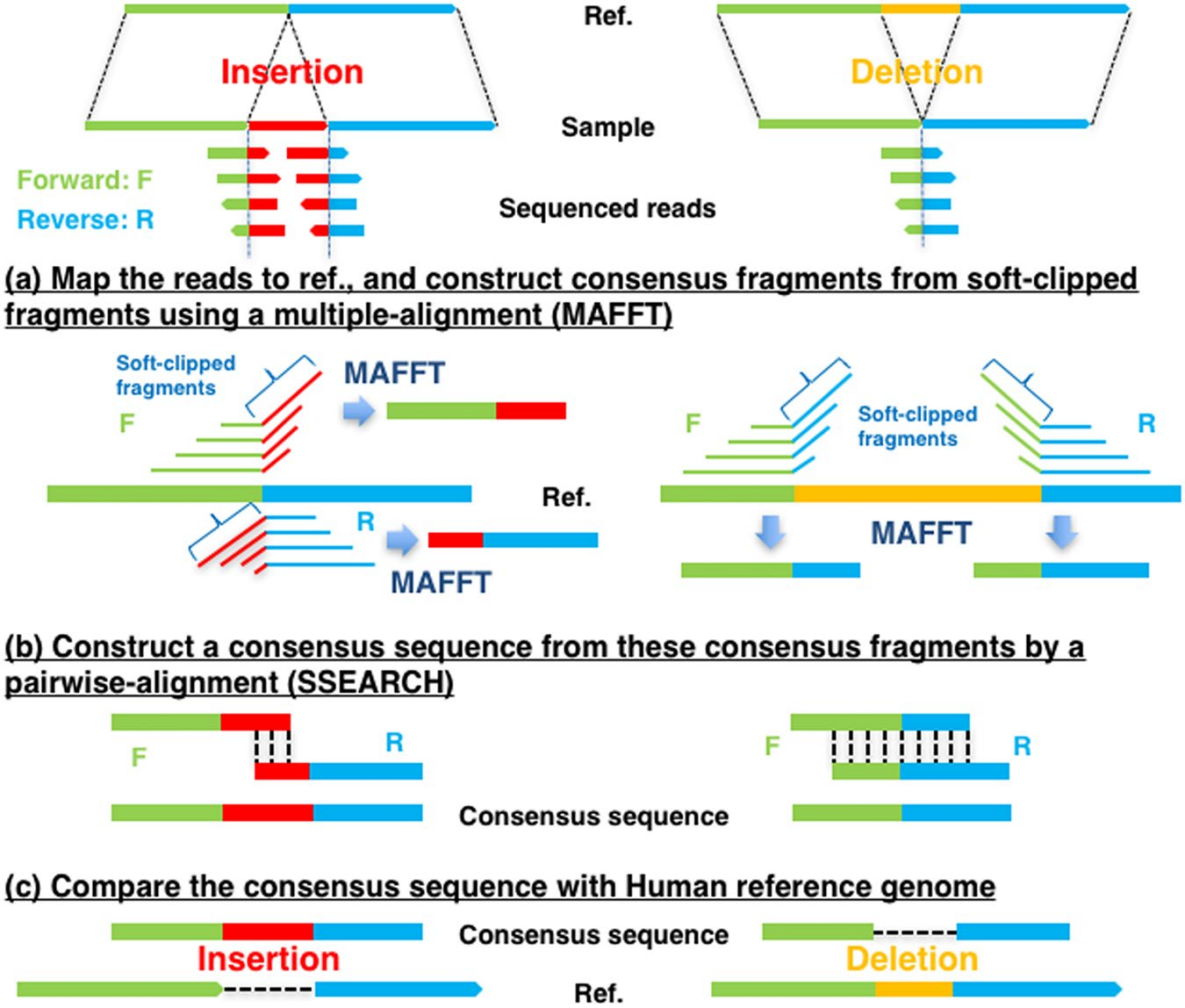
The workflow of intermediate-size indel prediction.

After mapping to the reference genome, reads were classified into three types: high quality soft-clipped reads, unmapped reads and mapped reads. The high quality soft-clipped fragments were further classified according to the position of the breakpoint (within 3 bp). We first constructed consensus fragments from the soft-clipped fragments and unmapped reads with mapped mates using multiple-alignments (Fig. 1a). In NA18943, 10,778 and 11,004 consensus fragments were constructed from 45,240 and 46,084 high quality soft-clipped fragments in forward and reverse orientation, respectively (Table 1). Next, we constructed consensus sequences using a pairwise sequence alignment of the mate pair consensus fragments (Fig. 1b). Through comparison of the consensus sequence and the reference sequence (5,000 bp upstream/downstream region from the breakpoint on the strand of the forward consensus fragment), we detected 60 intermediate-size indels with a total read depth ≥10 and a length between 50 bp and 10,000 bp. (Fig. 1c). In NA18948, 9,296 and 9,804 consensus fragments were constructed from 40,204 and 42,101 high quality soft-clipped fragments in the forward and reverse orientation, respectively (Table 1). Ultimately, 47 intermediate-size indels were detected. For NA12878, 1,179 and 1,283 consensus fragments were constructed from 7,062 and 6,826 high quality soft-clipped fragments in the forward and reverse orientation, respectively, and 17 intermediate-size indels were detected (Table 1).

**Table 1:** Summary for our intermediate-size indel prediction.

Sample	High quality soft-clipped fragments		Consensus fragment		Intermediate-size indel
	Forward	Backward	Forward	Backward	
NA18943	45,240	46,084	10,778	11,004	60
NA18948	40,204	42,101	9,296	9,804	47
NA12878	7,062	6,826	1,179	1,283	17

### Evaluation of IMSindel

We evaluated intermediate-size indel candidates predicted by the IMSindel, all of which were checked using Sanger sequencing of the NA18943 and NA18948 samples. Of the 60 and 47 candidates, 6 and 11 could not be amplified by PCR, respectively. Attempts to amplify these with a lower annealing temperature also failed. Of the amplified 54 and 36 candidates, 49 and 32 were consistent with our IMSindel genotype calls, respectively. The remaining 5 and 4 candidates were false positives. Of the 49 and 32 true positives, 37 (0.65) and 18 (0.56) were deletions in NA18943 and NA18948. In NA12878, PacBio long read sequencing data was used for the validation of the 17 predicted indels, of which one was a false positive (for details see Materials and Methods). Of the 16 true positives, 14 (0.88) were deletion. The precision (positive predictive value) was 0.91 (49/54) in NA18943, 0.89 (32/36) in NA18948 and 0.94 (16/17) in NA12878 (Table 2).

**Table 2:** Accuracy estimation of four call methods.

Sample		Genotype calls	^†^Sanger examined	TP (a)	FP (b)	FN (c)	Precision (a)/(a + b)	Recall (a)/(a + c)	F-measure
NA18943	IMSindel	60	54	49	5	14	0.91	0.78	0.84
	GATK	39	30	26	4	37	0.87	0.41	0.56
	PINDEL	70	60	32	28	31	0.53	0.51	0.52
	ScanIndel	32	24	20	4	43	0.83	0.32	0.46
NA18948	IMS	47	36	32	4	22	0.89	0.59	0.71
	GATK	17	15	15	0	39	1.00	0.28	0.43
	PINDEL	65	49	30	19	24	0.61	0.56	0.58
	ScanIndel	40	27	19	8	35	0.70	0.35	0.39
NA12878	IMSindel	17	—	16	1	8	0.94	0.67	0.78
	GATK	15	—	7	8	17	0.47	0.29	0.36
	PINDEL	22	—	14	8	10	0.64	0.58	0.61
	ScanIndel	19	—	10	9	14	0.53	0.42	0.47

^†^The number of genotypes that could be examined using Sanger sequencing.

### Performance comparison among four calling methods

We compared the performance of our IMSindel with that of three popular alternative methods, GATK HaplotypeCaller^1^, PINDEL^12^, and ScanIndel^16^ using the same HapMap-JPT samples. GATK HaplotypCaller predicted 39 intermediate-size indel candidates in NA18943 and 17 candidates in NA18948. Of the 39 and 17 candidates, 9 and 2 could not be amplified by PCR, respectively. Of the amplified 30 and 15 candidates, 4 candidates were false positives in NA18943, and none were false positives in NA18948. Of the 26 and 15 true positives, 14 (0.54) and 7 (0.47) were deletions in NA18943 and NA18948. In NA12878, 8 of the 15 candidates were false positives. Of the 7 true positives, 5 (0.71) were deletion. The precision was 0.87 (26/30) in NA18943 and 1.00 (15/15) in NA18948 and 0.47 (7/15) in NA12878 (Table 2 and Table S1).

PINDEL predicted 70 candidates in NA18943 and 65 candidates in NA18948. Of the 70 and 65 candidates, 10 and 16 could not be amplified by PCR, respectively. Of the amplified 60 and 49 candidates, 28 and 19 candidates were false positives. Of the 32 and 30 true positives, 26 (0.81) and 25 (0.83) were deletions in NA18943 and NA18948. In NA12878, 8 of the 22 candidates were false positives. Of the 14 true positives, 13 were deletion. The precision was 0.53 (32/60) in NA18943, 0.61 (39/49) in NA18948 and 0.64 (14/22) in NA12878 (Table 2 and Table S1).

The ScanIndel predicted 32 candidates in NA18943 and 40 candidates in NA18948. Of the 32 and 40 candidates, 8 and 13 could not be amplified by PCR, respectively. Of the amplified 24 and 27 candidates, 4 and 8 candidates were false positives. Of the 20 and 19 true positives, 16 (0.80) and 16 (0.84) were deletions in NA18943 and NA18948. In NA12878, 10 of the 19 candidates were true positives. All of the 10 true positives were deletion. The precision was 0.83 (20/24) in NA18943, 0.70 (19/27) in NA18948 and 0.53 (10/19) in NA12878 (Table 2 and Table S1).

In addition to precision, we examined the recall (sensitivity) for performance comparison of these four methods. We hypothesized that false negatives could be estimated using all of the validated indels across the four methods. The recall was calculated based on the false negative and true positive counts (for details see Materials and Methods). The recalls of IMSindel, GATK HaplotypeCaller, PINDEL and ScanIndel were 0.78, 0.41, 0.51 and 0.32 in NA18943, 0.59, 0.28, 0.56 and 0.35 in NA18948, and 0.67, 0.29, 0.58 and 0.42 in NA12878, respectively (Table 2).

In order to assess the overall performance of these four methods, we used the F-measure, the harmonic mean of the recall and precision. The highest F-measure observed was 0.84 and achieved by IMSindel in NA18943, for which HaplotypeCaller achieved 0.56 and PINDEL achieved 0.52, and ScanIndel achieved 0.46. These mirrored the results obtained in NA18948 and NA12878, 0.71 and 0.78 in IMSindel, 0.43 and 0.36 in GATK HaplotypeCaller, 0.58 and 0.61 in PINDEL and 0.39 and 0.47 in ScanIndel (Table 2), demonstrating that our IMSindel was superior to the other three methods for detecting intermediate-size indels.

**Figure 2:**
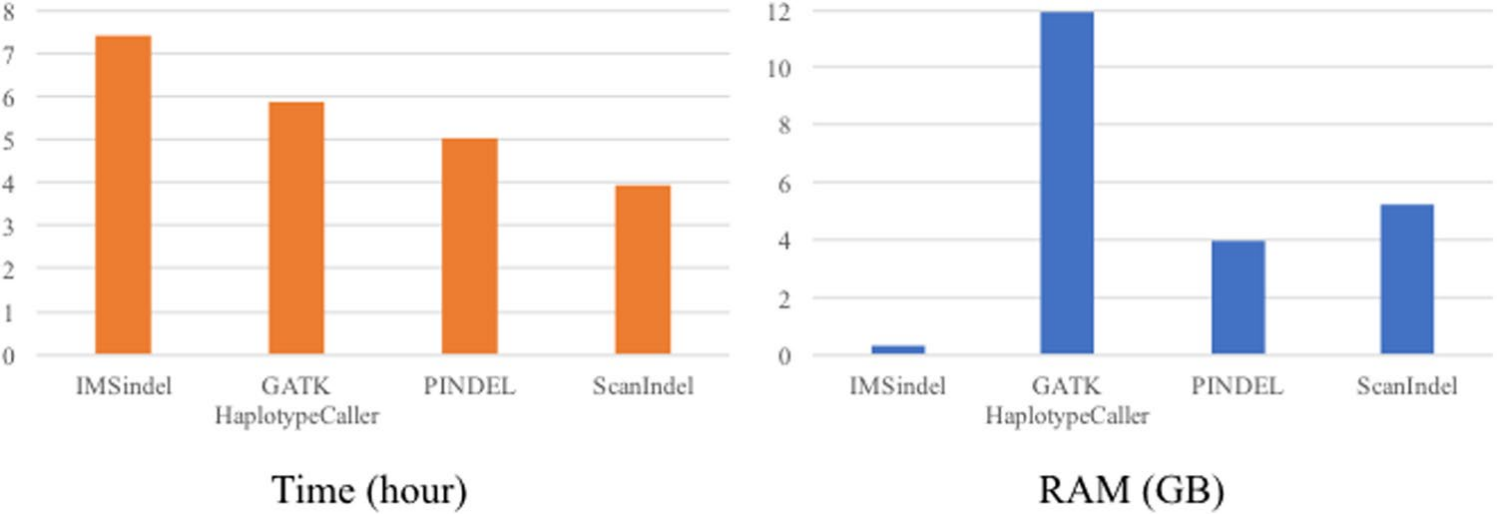
Time and peak memory used by four indel detection methods for NA18943.

We also compared the run time and memory usage of our IMSindel with the other methods when analyzing the high coverage WES data (NA18943) using 28-core Intel Xeon@2.40 GHz with 256 GB of memory. ScanIndel was the fastest indel detection method, which spent 3.9 hours to complete the analysis: IMSindel 7.4 hours, GATK HaplotypeCaller 5.9 hours, PINDEL 5.0 hours. IMSindel required the least memory (maximum 0.34 GB), likely because the indel detection is performed independently on each chromosome. GATK HaplotypeCaller required 11.9 GB, PINDEL required 4.0 GB, and ScanIndel required 5.2 GB (Fig. 2).

### Difference of intermediate-size indels among four methods

**Figure 3:**
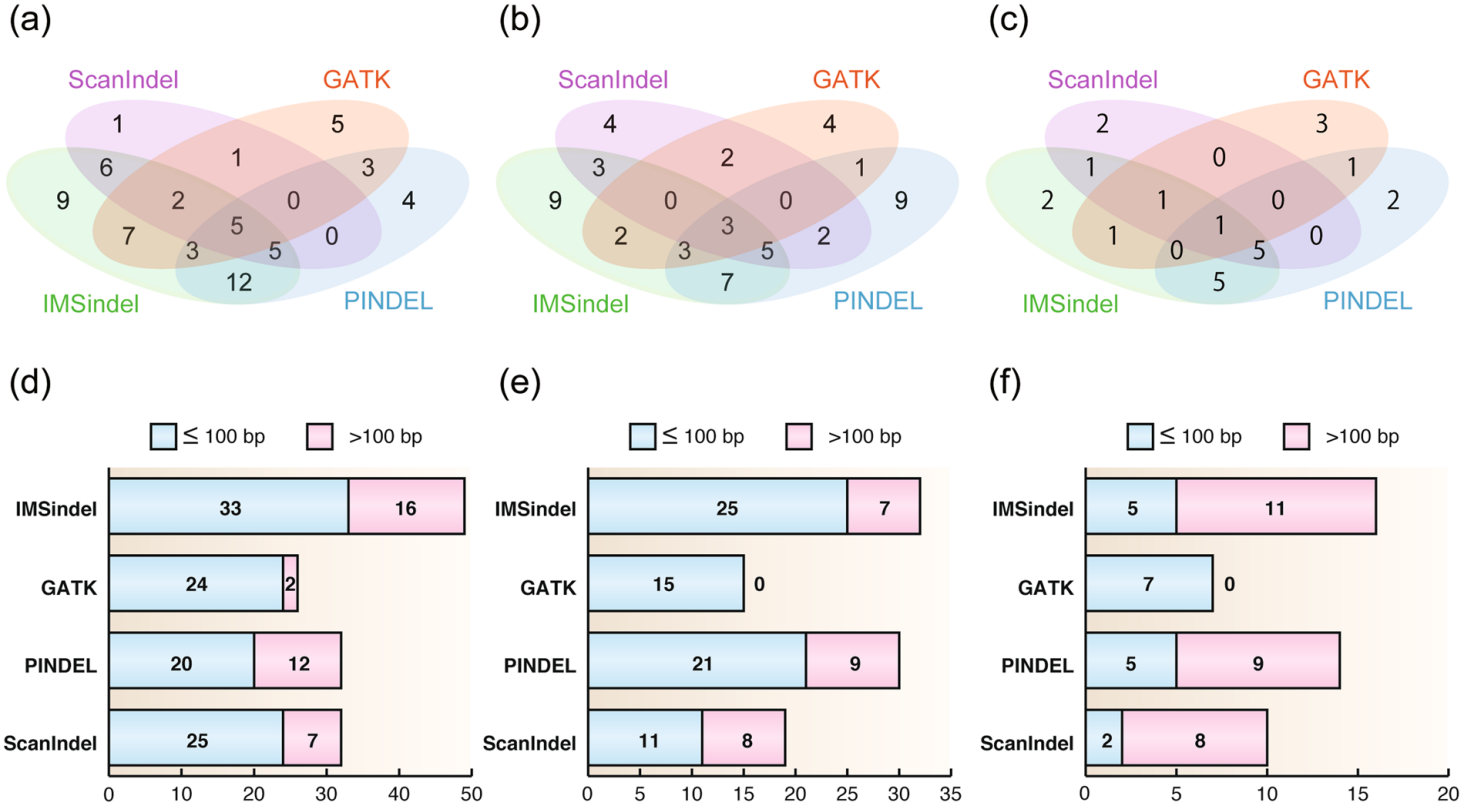
Intermediate-size indels detected by the three methods for NA18943. Venn diagram showing the overlap of the indels detected by all four methods: IMSindel, GATK HaplotypeCaller, PINDEL and ScanIndel in NA18943 (**a**), NA18948 (**b**) and NA12878 (**c**). The numbers of indel detected in the each method categorized by size in NA18943 (**d**), NA18948 (**e**) and NA12878 (**f**).

We examined the overlap of Sanger-validated indels detected in our IMSindel with those in the other methods. The majority of the indels were detected in at least two methods rather than in one method (common: 44, method-specific: 19 in NA18943, Fig. 3a; common: 28, method-specific: 26 in NA18948, Fig. 3b; common: 15, method-specific: 9 in NA12878, Fig. 3c). We further examined the distribution of the indel size among the four methods and found that IMSindel, PINDEL and ScanIndel detected many more indels greater than 100 bp than GATK HaplotypeCaller (GATK = 2, ScanIndel = 8, PINDEL = 12, IMSindel = 16 in NA18943, Fig. 3d; GATK = 0, ScanIndel = 8, PINDEL = 9, IMSindel = 7 in NA18948, Fig. 3e; GATK = 0, ScanIndel = 8, PINDEL = 9, IMSindel = 11 in NA12878, Fig. 3f).

### Distribution of intermediate-size indels predicted in IMSindel

**Figure 4:**
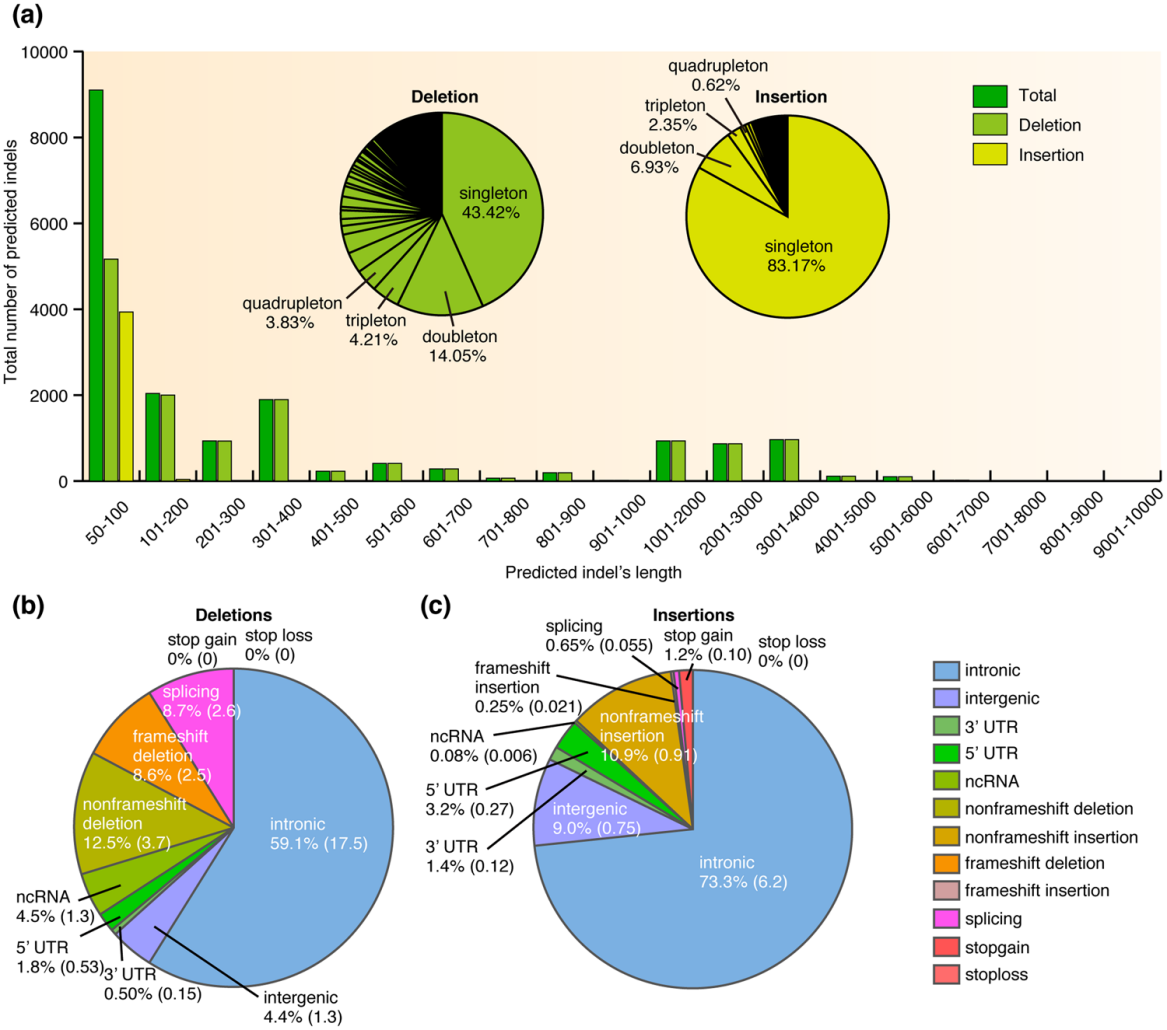
Distribution of intermediate-size indels predicted in IMSindel. (**a**) The total number of deletions and insertions predicted in 478 WES data. The percentage of 12 functional groups in predicted deletions (**b**) and insertions (**c**). The number in parenthesis indicates the number of predicted indels per sample.

We investigated intermediate-size indels predicted in IMSindel using human DNA samples from a consortium for congenital neurological diseases and hearing loss. We applied IMSindel to 478 WES datasets sequenced on the same whole exome sequencing platform. In total, 18,192 indels were predicted, of which 14,216 (0.78) were deletions and 3,976 (0.22) were insertions. These could be reduced to a unique set of 783 deletions and 808 insertions of different sizes. Of the 783 deletions and 808 insertions, 340 (0.43) and 672 (0.83) were singletons. Most of the indels were either singletons or doubletons (Fig. 4a). Although the size of many of the deletions and insertions were less than 100 bp, more long deletions were predicted than insertions. The longest deletion predicted was 6,546 bp and the longest insertion predicted was 213 bp (Fig. 4a).

We further classified these predicted indels into 12 functional groups (intronic, intergenic, UTR3, UTR5, ncRNA, nonframeshift deletion, nonframeshift insertion, splicing, frameshift deletion, frameshift insertion, stop gain and stop loss). In particular, we focused on the 5 groups (ncRNA, splicing, frameshift insertion, frameshift deletion, stop gain and stop loss) most affecting gene function and most likely to have a biological impact. Approximately 21.8% of the predicted deletions (Fig. 4b) and 2.2% of the insertions (Fig. 4c) were of these functionally important groups, with an average of 6 indels per sample (Fig. 4b and c). These results suggested that one of these functionally important indels may be disease-causing mutations, although these indels were not found in known disease genes.

### Performance comparison among call methods using simulation data

We compared the performance of four methods using simulation data. The simulation data sets were constructed by randomly placing 100 insertions and 100 deletions on human chromosome 22. The size of placed indels ranged from 100 bp to 1,000 bp at intervals of 100 bp. The sequence reads were generated with several parameters: point mutation rate (0.001 and 0.005), read length (75 bp and 150 bp), and sequencing coverage (100× and 200×) (for details see Materials and Methods).

**Figure 5:**
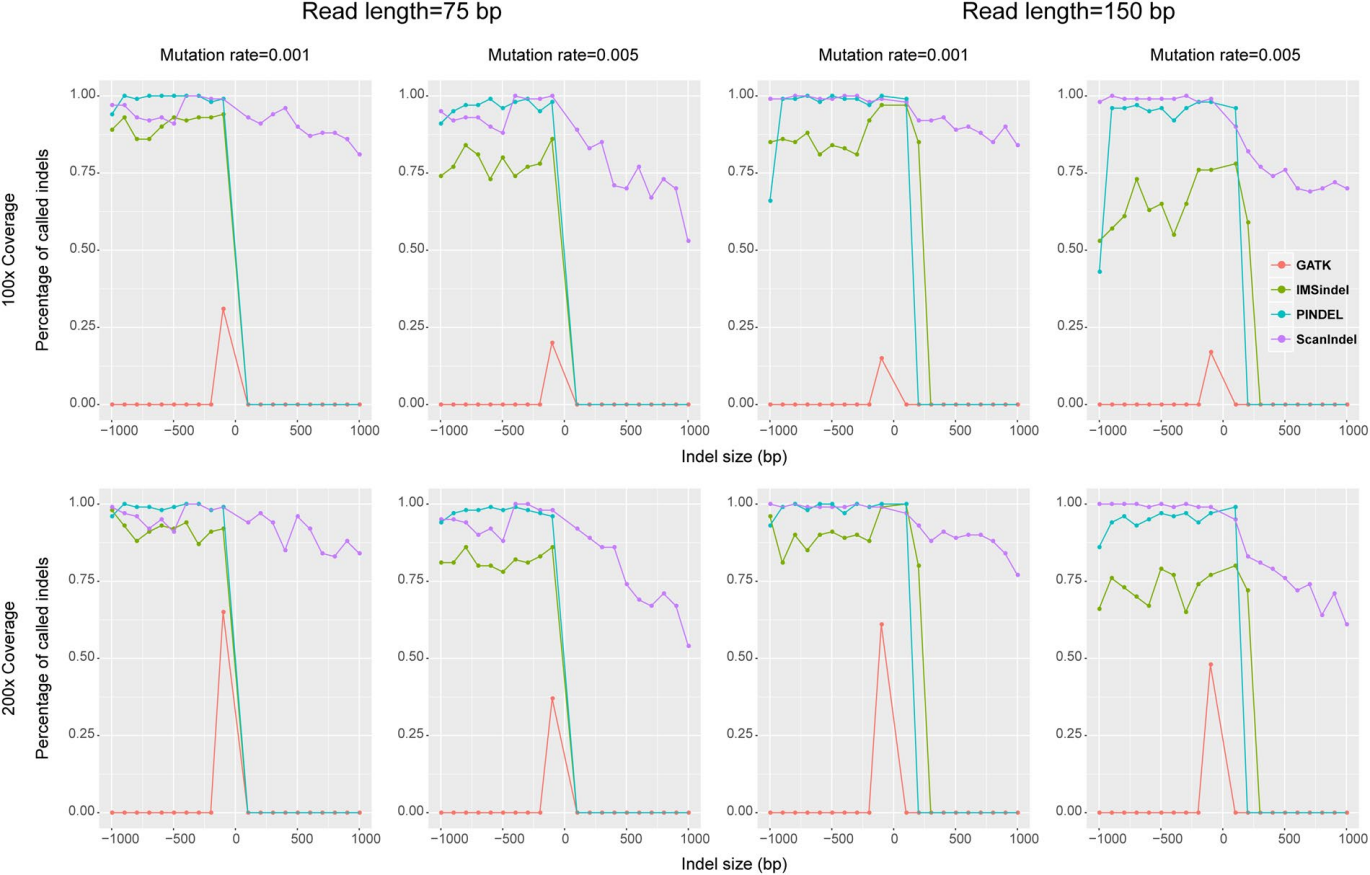
Performance comparison for indel detection using simulation data. The indel size ranged from 100 bp to 1,000 bp at interval 100 bp. Their sequence reads were generated with several parameters: point mutation rate (0.001 and 0.005), read length (75 bp and 150 bp), and sequencing coverage (100× and 200×).

Mapping of the simulation data was performed using BWA-MEM^9^. The same mapped read files were used for subsequent performance comparisons among the four methods. For deletions, all methods except for GATK HaplotypeCaller could successfully detect indels regardless of the size, although IMSindel was more sensitive to high mutation rates than the other methods. For insertions, all methods except for ScanIndel displayed only limited capability to detect them, although IMSindel performed better than PINDEL at longer read lengths (Fig. 5). Consequently, ScanIndel performed best in using these simulation data. There are two possible causes for the observed difference in performance with simulated and real data: (1) the concordance rates in real human WES data were calculated with respect to correct genotype, which would be very important in pedigree analysis, whereas the simulated set did not have a defined genotype, (2) *de novo* assembly does not perform as well in real human WES data due to many of the intermediate-size indels being located near or in repetitive elements. To further investigate the latter cause, we examined intermediate-size insertions detected in CDS in real human WES data, all of which were validated using Sanger sequencing. Out of 20 insertions, 14 shared similar or repetitive sequence with the flanking regions. Scanindel was able to detect the remaining 6 insertions (Table S2). We also evaluated these results using simulation data set by setting the insertion sequence to match repetitive elements in the flanking region. The sequence reads were generated with the parameters: point mutation rate (0.001), read length (150 bp), and sequencing coverage (200×). We found that when the insertions were ≥2  bp length and contained repetitive sequence from the flanking region, *de novo* assembly (Inchworm) did not work well (Table S3).

## Discussion

To identify causative genetic mutations of disease, whole exome sequencing (WES) is widely used^17^. Several WES analyses have recently succeeded in identifying causal mutations of Mendelian diseases^18,19^. However, the reported detection rates for the deleterious mutations range from 25% to 50%^20,21^. Mutations have not yet been detected in many patients analyzed. One of the reasons why these mutations were not detected from WES data might be due to the standard WES analysis approach, which considers only single nucleotide variants (SNVs) and short indels. In other words, since typical WES analysis often does not investigate intermediate-size indels, our method may identify disease-causing mutations previously missed.

Several sophisticated computational approaches have been developed to accurately detect SNPs and short indels ( < 50 bp) from next-generation sequencing (NGS) data^1–3^. However, accurate detection of the intermediate-size indels (50 bp to 10,000 bp) from NGS data is still challenging due to the typically short length of DNA sequencing reads. Although several algorithms, such as GATK HaplotypeCaller^1^, PINDEL^12^, and ScanIndel^16^, attempt to predict these intermediate-size indels, these methods lack concordance when applied to real human NGS data sets^22^. Therefore, we developed a new accurate method for predicting these intermediate-size indels using BWA soft-clipped fragments (unmatched fragments in partially mapped reads) and unmapped reads. False positive and false negative rates were determined through Sanger sequencing of all predicted indels across these three methods and ours (IMSindel). We demonstrated that our method was more accurate and applicable than the current popular alternative methods for genotype calling of the intermediate-size indels in real human WES data, although ScanIndel had best performance in simulation data. Possible explanations of these differences are: (1) the concordance rates in real human WES data were calculated with respect to correct genotype, which would be very important in pedigree analysis, whereas the simulated set did not have a defined genotype, (2) *de novo* assembly does not work well in real human WES data, where many of the intermediate-size indels are located in or near repeat content.

Finally, our method was able to detect several intermediate-size indels per sample that were likely to affect gene function when applied to a large number of real human WES data. Our results suggest that our method could be a new approach to detect deleterious mutation associated with disease. However, since we hypothesized that false negatives could be estimated using all of the validated indels across the four methods, the recalls calculated using the false negatives could be overestimated.

In this study, we present IMSindel as a robust method for more accurate prediction of intermediate-size indels from real human WES data. While we successfully applied IMSindel to germline datasets with WES, IMSindel can also be extended to WGS data analysis. We believe that this methodology will contribute to the discovery of deleterious mutations associated with human diseases in near future.

## Materials and Methods

### Ethics Statement

This study was approved by the ethics committee of Tokyo Medical and Dental University, RIKEN, Osaka National Hospital, Osaka Medical Center and Research Institute for Maternal and Child Health, Nagoya City University Graduate School of Medical Sciences, Showa University School of Medicine, Takatsuki General Hospital, and National Hospital Organization Tokyo Medical Center. The design and performance of the current study involving human subjects were clearly described in a research protocol. All participants were voluntary and would complete the informed consent in written before taking part in this research. All the methods were performed in accordance with the relevant guidelines and regulations.

### DNA Sample

The HapMap-JPT samples (NA18943, NA18948 and NA12878) were obtained from Coriell, where lymphoblastoid cell lines were established by Epstein-Barr virus (Human herpesvirus 4)-mediated transformation of peripheral blood mononuclear cells. The samples were used for the accuracy evaluation of our method, GATK HaplotypeCaller, PINDEL and ScanIndel. For real case performance of our method, we also used 478 DNA samples collected from a consortium for congenital neurological diseases and hearing loss after obtaining written informed consent^23^.

### Whole-exome sequencing

The Agilient SureSelect Human All Exon V5 was used for exome capture for two DNA samples (NA18943 and NA18948) according to the manufacturer’s instructions. These kits capture genomic DNA by in-solution hybridization with RNA oligonucleotides, enabling specific targeting of approximately 51 Mb of the human genome. The captured DNA was sequenced using the Illumina HiSeq. 2500 platform with paired-end reads of 101 bp or 161 bp according to the manufacturer’s instructions. For NA12878, WES data with paired-end reads was used (SRR098401), available from ftp://ftp.1000genomes.ebi.ac.uk/vol1/ftp/phase3/data/NA12878/sequence_read/. The PacBio long read sequencing data (NA12878.sorted.vcf.gz) was used for the validation of our predicted indels, available from ftp://ftp-trace.ncbi.nlm.nih.gov/giab/ftp/data/NA12878/NA12878_PacBio_MtSinai/. Also, indels commonly predicted in multiple methods were handled as true positives.

### Read mapping

Read sequences were mapped by the Burrows-Wheeler Aligner (BWA-MEM: version 0.7.15)^9^ to the human reference genome (GRCh37) with default parameters, as BWA-MEM supports long read and split-read alignment. The mapped reads were sorted using SAMtools (version 0.1.8)^24,25^, and duplicate PCR reads were subsequently identified and marked using the Picard tool (version 1.119) (http://broadinstitute.github.io/picard/).

### Intermediate-size indel prediction

#### Consensus fragment detection using multiple alignment

Based on the genome mapping data (SAM or BAM output), we classified read sequences into three types: high quality soft-clipped reads, unmapped reads and mapped reads. The high quality soft-clipped reads were based on an overall mapping quality >20 and a soft-clipped fragment average base quality >20 with a length >5. The high quality soft-clipped fragments were further classified according to sharing a breakpoint within 3 bp. We used unmapped reads with mapped mates for construction of consensus fragments from the soft-clipped fragments using a multiple sequence alignment program (MAFFT^26^, Fig. 1a). These breakpoints of the unmapped reads were estimated using the mapped mate pairs and insert sizes for paired-end sequencing. These unmapped reads contribute to the detection of intermediate-size insertion. This command used for the MAFFT allowing large gaps was “mafft–nuc–ep 0.0–op 1–genafpair–maxiterate 1000 input_file”.

#### Consensus sequence detection using global-local pairwise alignment

Using a pairwise sequence alignment, we constructed a consensus sequence from mate pair consensus fragments (Fig. 1b). An optional global-local search of FASTA programs^27,28^, ‘*glsearch*’, was applied to the pairwise alignment, and the scoring matrix used was set that mismatched alignments have a large penalties (20). Consensus sequences were identified with a following command; “glsearch36 -s mydna.mat -g0 -f20 consensus_fragment1 consensus_fragment2”.

#### Indel detection from the difference between a consensus sequence and a reference sequence

The reference sequence was defined as the 5,000 bp upstream/downstream region from the breakpoint on the strand of the forward consensus fragment (Fig. 1c). The reference sequence was compared to consensus sequence, and intermediate-size indels (50 bp to 10,000 bp) were detected with a following command; “mafft–nuc–ep 0.0–op 1–genafpair–maxiterate 1000 input_file” (Fig. 1c).

### Accuracy evaluation

In order to evaluate the accuracy of all three call methods (our method, GATK HaplotypeCaller and PINDEL), we validated all of the method-specific calls using Sanger sequencing and calculated the number of false positives (FP). The number of true positives (TP) was defined as the number of correctly predicted genotypes. The false negative (FN) was estimated from the performance comparison of all three methods. To assess the performance of each method, we used precision (positive predictive value), recall (sensitivity) and F-measure as defined below:

1 $$Precision=\frac{TP}{TP+FP}$$

2 $$Recall=\frac{TP}{TP+FN}$$

3 $$F-measure=\frac{2\times Precision\times Recall}{Precision+Recall}$$

### Simulation data sets

To evaluate our method and compare it with the other intermediate-size indel detection methods, we generated in silico data. Human chromosome 22 (GRCh37) was used as the reference genome. First, we divided the reference genome into 10 kb bins, and randomly selected 100 bins for simulations. Next, we placed insertions and deletions ranging from 100 bp to 1,000 bp at interval 100 bp for the selected bins, respectively. These inserted and deleted sequences were randomly generated using svsim software available from https://github.com/GregoryFaust/SVsim.

To generate paired-end reads including these inserted or deleted sequences, we used wgsim software available from https://github.com/lh3/wgsim. To compare our method with the other methods in different sequencing conditions, we generated the reads with several parameters: point mutation rate (0.001 and 0.005), read length (75 bp and 150 bp), and sequencing coverage (100× and 200×). Also, the base error rate, the outer distance between paired-end reads, the standard deviation was then set to 0.02, 500 bp, and 50 bp without additional indel mutations, respectively.

## Electronic supplementary material

Table S1

Table S2

Table S3
