# Publisher Correction: IMSindel: An accurate intermediate-size indel detection tool incorporating *de novo* assembly and gapped global-local alignment with split read analysis

**DOI:** 10.1038/s41598-018-28698-y

**Published:** 2018-07-04

**Authors:** Daichi Shigemizu, Fuyuki Miya, Shintaro Akiyama, Shujiro Okuda, Keith A. Boroevich, Akihiro Fujimoto, Hidewaki Nakagawa, Kouichi Ozaki, Shumpei Niida, Yonehiro Kanemura, Nobuhiko Okamoto, Shinji Saitoh, Mitsuhiro Kato, Mami Yamasaki, Tatsuo Matsunaga, Hideki Mutai, Kenjiro Kosaki, Tatsuhiko Tsunoda

**Affiliations:** 1 Department for Medical Genome Sciences, Medical Genome Center, National Center for Geriatrics and Gerontology, Aichi, Japan; 2 Department of Medical Science Mathematics, Medical Research Institute, Tokyo Medical and Dental University (TMDU), Tokyo, Japan; 3 RIKEN Center for Integrative Medical Sciences, Yokohama, Japan; 4 Medical Sciences Innovation Hub Program, Cluster for Science and Technology Hub, RIKEN, Yokohama, Japan; 5 CREST, JST, Japan; 6 Niigata University Graduate School of Medical and Dental Sciences, Niigata, Japan; 7 Department of Drug Discovery Medicine, Graduate School of Medicine, Kyoto University, Kyoto, Japan; 8 Division of Regenerative Medicine, Institute for Clinical Research, Osaka National Hospital, National Hospital Organization, Osaka, Japan; 9 Department of Neurosurgery, Osaka National Hospital, National Hospital Organization, Osaka, Japan; 10 Department of Medical Genetics, Osaka Medical Center and Research Institute for Maternal and Child Health, Osaka, Japan; 11 Department of Pediatrics and Neonatology, Nagoya City University Graduate School of Medical Sciences, Nagoya, Japan; 12 Department of Pediatrics, Showa University School of Medicine, Tokyo, Japan; 13 Department of Pediatric Neurosurgery, Takatsuki General Hospital, Osaka, Japan; 14 Division of Hearing and Balance Research, National Institute of Sensory Organs, National Hospital Organization Tokyo Medical Center, Tokyo, Japan; 15 Center for Medical Genetics, Keio University School of Medicine, Tokyo, Japan

Correction to: *Scientific Reports* 10.1038/s41598-018-23978-z, published online 04 April 2018

This Article contains an error in the Results section under the subheading ‘Performance comparison among call methods using simulation data’.

“We found that when the insertions were ≥2 bp length and contained repetitive sequence from the flanking region, *de novo* assembly (Inchworm) did not work well (Table S3).”

should read:

“We found that when the insertions were ≥25 bp length and contained repetitive sequence from the flanking region, *de novo* assembly (Inchworm) did not work well (Table S3).”

